# Timeline of COVID-19 Incidence and Mortality among Residents and Staff of South Carolina Long-term Care Facilities

**DOI:** 10.1093/geroni/igab046.2713

**Published:** 2021-12-17

**Authors:** Matthew Lohman, Nicholas Resciniti, Morgan Fuller, Joshua Sellner

**Affiliations:** 1 University of South Carolina, Columbia, South Carolina, United States; 2 University of Southern California, Los Angeles, California, United States; 3 South Carolina Department of Health & Environmental Control, Columbia, South Carolina, United States

## Abstract

The COVID-19 pandemic has disproportionately impacted older adults living in long-term care facilities (LTCFs), but little research has described parallel infection rates and mortality among LTCF residents and staff in relation to state-level mitigation measures. This study used comprehensive COVID-19 tracking data from the South Carolina Department of Health and Environmental Control (SCDHEC), including case report information on demographics, symptoms, comorbidities, and employment. We included all confirmed or probable COVID-19 cases and deaths among adult SC residents reported between 3/15/2020 and 1/2/2021. Residence or employment in LTCF, including nursing homes, assisted living, or skilled nursing facilities, were confirmed by SCDHEC. Cox proportional hazards models were used to compare mortality between residents/staff and counterparts in the community. Overall, 54,514 cases of COVID-19 were identified among older adults in SC. Of these, 13.5% (n = 7,366) resided in a LTCF. LTCF residents with COVID-19 were more likely to be hospitalized compared to older adults in the community and 74% more likely to die (HR= 1.74, 95% CI: 1.59-1.90), after controlling for age, gender, race, and chronic health conditions. LTCF staff had greater infection rates but lower risk of mortality (HR=0.58; 95% CI: 0.39-0.88) compared to the general population. Differences in COVID-19 incidence and mortality between residents/staff and the community decreased after statewide mitigation policies. This study indicates that LTCF residents are at increased risk of COVID infection and mortality, even accounting for pre-existing health conditions. LTCF settings are key sites for prioritizing prevention, vaccination, and training plans to prepare for future pandemics.

